# Content validity and psychometric evaluation of Functional Assessment of Chronic Illness Therapy-Fatigue in patients with psoriatic arthritis

**DOI:** 10.1186/s41687-019-0115-4

**Published:** 2019-05-20

**Authors:** David Cella, Hilary Wilson, Huda Shalhoub, Dennis A. Revicki, Joseph C. Cappelleri, Andrew G. Bushmakin, Elizabeth Kudlacz, Ming-Ann Hsu

**Affiliations:** 1 Department of Medical Social Sciences, Northwestern University, Chicago, IL USA; 2 Evidera, Bethesda, MD USA; 3 Evidera, Waltham, MA USA; 4 Pfizer Inc, Groton, CT USA

**Keywords:** FACIT-Fatigue, Psoriatic arthritis, Content validity, Tofacitinib, Psychometric properties

## Abstract

**Background:**

To evaluate the measurement properties (e.g., content validity, reliability, and ability to detect change) of the Functional Assessment of Chronic Illness Therapy (FACIT)-Fatigue scale in patients with active psoriatic arthritis (PsA).

**Methods:**

One-on-one semi-structured qualitative interviews with adult patients with active PsA evaluated the content validity of FACIT-Fatigue. Quantitative measurement properties were evaluated using data from phase III tofacitinib randomized controlled trials (RCTs) in PsA: OPAL Broaden (NCT01877668) and OPAL Beyond (NCT01882439).

**Results:**

Of 12 patients included in the qualitative study, 2 (17%) had mild, 8 (67%) had moderate, and 2 (17%) had severe PsA disease activity; 7 (58%) attributed fatigue to PsA, and 7 (58%) rated fatigue as important or extremely important. Most patients considered the FACIT-Fatigue items relevant to their PsA experience, and understood item content and response options as intended. In the psychometric analysis of RCT data, a second-order confirmatory factor model fit the data well (Bentler’s Comparative Fit Index ≥0.92). FACIT-Fatigue demonstrated good internal consistency (Cronbach’s coefficient *α* ≥ 0.90), test-retest reliability (Intraclass Correlation Coefficient ≥ 0.80) and a strong correlation with SF-36 Vitality (*r* > 0.80). A robust relationship between disease activity (based on Patient’s Global Assessment of Psoriasis and Arthritis) and FACIT-Fatigue was observed (effect sizes > 1.4), with clinically important difference for the FACIT-Fatigue total score estimated as 3.1 points, and the responder definition estimated as a 4-point improvement for FACIT-Fatigue total score.

**Conclusion:**

Fatigue was confirmed to be an important symptom to patients with PsA, and FACIT-Fatigue was found to be a reliable and valid measure in this population.

**Electronic supplementary material:**

The online version of this article (10.1186/s41687-019-0115-4) contains supplementary material, which is available to authorized users.

## Background

Psoriatic arthritis (PsA) is a chronic inflammatory disease occurring in 6–42% of patients with psoriasis [1]. It is characterized by joint inflammation, enthesitis, dactylitis, and spondylitis, and is often associated with generalized fatigue [1–3].

Fatigue was recently added to the core domain set for PsA randomized controlled trials (RCTs) [4, 5], due to the impact that it has on a patient’s quality of life. Patients with PsA have noted statistically significant improvements in fatigue following treatment with newer agents such as certolizumab, secukinumab, and apremilast [6–8], suggesting it is modifiable with treatment. For example, in patients with PsA, intravenous secukinumab 150 mg led to a least squares mean change from baseline in fatigue of 6.74 (*P* < 0.05 vs. placebo), as measured by the Functional Assessment of Chronic Illness Therapy-Fatigue (FACIT-Fatigue) scale [8].

Although recognized as a core domain for assessment in RCTs, there is currently no universally accepted measure of fatigue recommended to evaluate this construct in patients with PsA. When measuring a construct within the RCT setting, it is important to ensure the relevance and comprehension of a questionnaire to the target population, and its reliability, validity, and ability to detect change [9–11].

The FACIT-Fatigue scale [12] (Additional file 1: Appendix 1: Figure S1) is a 13-item questionnaire originally designed to assess fatigue/tiredness and its impact on daily functioning in people with cancer; it has now been evaluated in other chronic diseases [12–15]. Each item’s response option uses a 5-point scale ranging from “not at all” to “very much.” The total FACIT-Fatigue score ranges from 0 to 52, where higher scores represent less fatigue [13, 14]. While commonly applied as one overall score, previous work has shown that the measurement model of the FACIT-Fatigue scale includes two distinguishable domains, representing the impact and experience of fatigue, in addition to the global domain (represented by the overall score) [16].

Psychometric data in patients with RA suggest that FACIT-Fatigue (total score; baseline, Week 12, and Week 24 assessments) has good internal consistency (*α* = 0.86 to 0.87) and the ability to differentiate patients according to clinical change using the American College of Rheumatology response criteria, [12]. FACIT-Fatigue also showed a strong association with the longer, 16-item Multidimensional Assessment of Fatigue scale (*r* = − 0.84 to − 0.88), implying a redundancy between these two measures. However, this study also reported that FACIT-Fatigue captured a broader distribution of patients and wider range of self-reported fatigue concepts. A qualitative study of 17 patients with moderate to highly active RA found FACIT-Fatigue to have high content validity; 10 of the 13 items had “high” content validity (determined by the relationship between the intended measurement concept and the methods used [17]), with three having “low to moderate” (“I feel weak all over”, “I feel listless [washed out]”) or “low” (“I am too tired to eat”) content validity [18]. This study also concluded that FACIT-Fatigue captured most fatigue-related, patient-reported concepts. Chandran and colleagues also showed FACIT-Fatigue to have good internal consistency (*α* = 0.96) and significant correlation with actively inflamed joint count (*r* = − 0.43) in patients with PsA [14]. However, there is currently no qualitative evidence to support content validity in patients with PsA, and no quantitative evidence supporting other measurement properties specifically in an RCT.

We designed a mixed-methods approach to further evaluate the qualitative and quantitative measurement properties of FACIT-Fatigue in patients with PsA. For the former, a qualitative study was designed to: 1) elicit concepts important to patients with PsA regarding the signs, symptoms, and impact of PsA on daily functioning, focusing on the experience and impact of fatigue; and 2) evaluate the content validity of the FACIT-Fatigue scale. For the latter, a secondary analysis of two phase III RCTs of tofacitinib assessed FACIT-Fatigue in patients with moderate to severe PsA.

## Patients and methods

### Qualitative FACIT-Fatigue study

Combined concept elicitation and cognitive interviews were carried out prior to the quantitative analysis and included one-on-one semi-structured interviews with 12 adult patients (aged ≥18 years) who had a confirmed diagnosis and presence of active PsA [19] (full details in Additional file 2: Appendix 2a). Interviews were conducted in-person at two clinical sites in the United States (Florida and Pennsylvania), by two experts (research associates, Evidera) who were trained and experienced in qualitative interviewing methods. The sample size of the qualitative study was determined by an estimated projection of saturation [20, 21] based on previous experience with clinical outcome assessment content validation research and the literature [10, 22].

### Patient interviews

Prior to the start of each interview, the interviewers fully explained the study to the patient and obtained written, informed consent. Interviewers led the discussion using a standardized, semi-structured interview guide (full guide in Additional file 2: Appendix 2b), divided into two parts. Part 1, an open-ended concept elicitation, was designed to assess relevant symptom and impact concepts (e.g., self-reported PsA severity), and understand the relative importance and patients’ experience of fatigue. If patients did not spontaneously report signs or symptoms of their PsA, the interviewer probed further, in line with the interview guide (Additional file 2: Appendix 2b). Detailed questions related to fatigue were followed by general questions about patients’ overall symptoms and impact on functioning.

In part 2, patients completed the FACIT-Fatigue questionnaire and were asked to provide feedback on overall comprehension and relevance. Questions were designed to assess the interpretation of instructions, items, the recall period, and the response options. Following the interview, patients completed a sociodemographic and clinical questionnaire. Qualitative data were then analyzed using ATLAS.ti qualitative data analysis software version 7.5.15 [23], using a coding dictionary and thematic analysis techniques [10, 24–26, 20] (further information provided in Additional file 2: Appendix 2a).

### Psychometric analysis of FACIT-Fatigue in PsA

Subsequently to the qualitative assessment, a series of analyses assessed the quantitative psychometric properties of the FACIT-Fatigue scale, based on data from the phase III RCTs OPAL Broaden (NCT01877668) [27] and OPAL Beyond (NCT01882439) [28]. These analyses were pre-specified in a psychometric statistical analysis plan.

OPAL Broaden was a 12-month RCT in patients with an inadequate response to ≥1 conventional synthetic disease-modifying antirheumatic drug (csDMARD) and who were tumor necrosis factor inhibitor (TNFi)-naive. Patients (*n* = 422) were randomized 2:2:2:1:1 to tofacitinib 5 mg twice daily (BID; *n* = 107), tofacitinib 10 mg BID (*n* = 104), adalimumab 40 mg once every 2 weeks (*n* = 106), placebo advancing to tofacitinib 5 mg BID at Month 3 (*n* = 52), or placebo advancing to tofacitinib 10 mg BID at Month 3 (*n* = 53) [27]. OPAL Beyond was a 6-month RCT in patients who had an inadequate response to ≥1 TNFi (TNFi-IR). Patients (*n* = 395) were randomized 2:2:1:1 (394 patients received treatment) to tofacitinib 5 mg BID (*n* = 131; one patient randomized but not treated), tofacitinib 10 mg BID (*n* = 132), placebo advancing to tofacitinib 5 mg BID at Month 3 (*n* = 66), or placebo advancing to tofacitinib 10 mg BID at Month 3 (*n* = 65) [28]. In both RCTs, patients received a stable background dose of one csDMARD.

FACIT-Fatigue data from both RCTs were pooled across all treatment groups to provide the largest sample size and response range to the individual items. Two different pooling strategies were used. Strategy 1: Pooled Data 1 (PD1; OPAL Beyond baseline data pooled with OPAL Broaden Month 12 [last study visit]; number of observations = 760, one observation per patient) and Pooled Data 2 (PD2; OPAL Broaden baseline data pooled with OPAL Beyond Month 6 [last study visit]; number of observations = 766, one observation per patient) were used in the cross-sectional analyses (i.e., internal consistency reliability, confirmatory analyses, and correlations). Strategy 2: for longitudinal analyses (i.e., test-retest, clinically important difference [CID], and responder definition [RD]), Pooled Data 3 (PD3) was used, corresponding to all available data from OPAL Broaden pooled longitudinally with all available data from OPAL Beyond.

#### Confirmatory factor analysis model

The FACIT-Fatigue measurement model was based on the conceptual framework and was represented by a second-order confirmatory factor analysis. This measurement model was evaluated using PD1 and PD2 and included the two FACIT-Fatigue scale scores and the total score. It was assumed that the latent construct “Experience” (represented by the first-order factor f1) affects items 1, 2, 3, 4, and 7 of FACIT-Fatigue and the latent construct “Impact” (represented by the first-order factor f2) affects all other nine items. The latent aggregated factor (represented by the second-order factor f3) affects “Experience” and “Impact” domains (Additional file 2: Appendix 2c, Figure S2 and factor loadings shown in Figure S3).

Bentler’s Comparative Fit Index (CFI) was used to measure the fit of the model with the data. An acceptable fit was defined as: 1) CFI > 0.90; 2) unstandardized path coefficients are statistically significant (*P* value < 0.05); and 3) standardized path coefficients are > 0.40 and are statistically significant.

Supplemental analyses using bifactor confirmatory factor modeling were also performed, where FACIT-Fatigue was represented by the global factor (latent factor fg; Additional file 2: Appendix 2c, Figure S4), and “Experience” and “Impact” domains were modeled as the group/nuisance factors (latent factors f1 and f2, respectively; Additional file 2: Appendix 2c, Figure S4).

#### Internal consistency reliability

Cronbach’s Coefficient *α* assessed internal consistency reliability of FACIT-Fatigue, with good internal consistency defined as a Cronbach’s coefficient *α* ≥ 0.90 (Additional file 2: Appendix 2d, Figure S5 details FACIT-Fatigue conceptual framework).

#### Test-retest reliability

Intraclass Correlation Coefficients (ICC) estimated test-retest reliability using baseline and Month 1 data. Because of the treatment intervention, a subgroup of “stable” patients was used in the analysis, with an ICC ≥ 0.70 defined as acceptable [29]. To define a stable subgroup, the Patient’s Global Assessment (PtGA; a component of the Patient’s Global Joint and Skin Assessment) was used. PtGA was formulated as follows: “In all the ways in which your psoriasis and arthritis, as a whole, affects you, how would you rate the way you felt over the past week?”. PtGA is a Visual Analog Scale (VAS) from 0 mm (poor) to 100 mm (excellent). To estimate ICC in this analysis, it was assumed that a less than 10 mm difference at Month 1 from baseline represents a “stable” patient.

#### Convergent validity

Evidence of convergent validity (the extent to which two concepts are related to one another [30]) was evaluated by correlation of the FACIT-Fatigue domain scores with other outcomes from the same studies (SF-36 domains, Itch Severity Item [ISI], Dermatology Life Quality Index [DLQI] total score, and Patient’s Global Assessment of Psoriasis and Arthritis [PtGA], Patient’s Skin Assessment [PtSA], and Patient’s Joint Assessment [PtJA], which are components of the Patient’s Global Joint and Skin Assessment – Visual Analog Scale [PtGJS-VAS]). Correlations of FACIT-Fatigue with these outcomes were expected to be ≥0.40, previously considered a moderate correlation [31].

#### Defining the clinically important difference for FACIT-Fatigue domains

Clinically important difference (CID), the difference in scores between two treatment groups that is considered clinically relevant, was estimated using a repeated measures model (RMM), assessing the relationship between the PtGA score and FACIT-Fatigue domains in PD3. The domain (Impact or Experience) of FACIT-Fatigue (including total score) is the outcome, and PtGA is a continuous or categorical anchor (RMM-CID). The SF-36 Vitality domain was also used as an anchor, in addition to being used in the sensitivity analyses.

When using PtGA as an anchor, it is important to note that it is a VAS; hence, there are no clear patient-selected categories to use as a basis to define a CID. To estimate a CID for PtGA, it is first assumed that the 100 mm VAS PtGA (used in OPAL Broaden and OPAL Beyond) can be linearly approximated by a 7-point scale (e.g., Patient Global Impression-Severity). From this, it can then be assumed that a value of 17 mm could be representative of the one-category difference and could be used to estimate the CID for a FACIT-Fatigue domain (note that 17 mm = 100 mm/6, where 6 is the number of pairwise adjacent categories) (further details in Additional file 2: Appendix 2a) [32, 33].

#### Defining the responder definition for FACIT-Fatigue domains

Responder definition (RD), the amount of change an individual patient would have to report to indicate that a relevant treatment benefit has been experienced, was estimated using a RMM to assess the relationship between a new anchor, the “Subject Global Impression of Change” (SGIC) score with just three categories (“better”, “the same”, and “worse”), and FACIT-Fatigue domains in PD3 (RMM-RD) (further details in Additional file 2: Appendix 2a).

#### Known-groups validity

Known-groups validity was evaluated based on a RMM-CID model by comparing FACIT-Fatigue scores between groups known to be different based on PtGA as the criteria. Ability to detect change was based on a RMM-CID model by examining the relationship between FACIT-Fatigue scores and PtGA. Patients were classified as “in remission/low disease” if they reported a score of 0 mm on the PtGA, and patients were classified as “active disease” if they reported a score of 100 mm.

Effect sizes were estimated by dividing the difference in score by standard deviation at baseline, and provide a general set of thresholds or benchmarks through adjectival descriptors on the difference between groups or impact of an intervention, with values of 0.2 generally regarded as “small,” 0.5 as “medium,” and 0.8 as “large”.

### Study oversight

OPAL Broaden (NCT01877668) [27] and OPAL Beyond (NCT01882439) [28] were conducted in accordance with the International Conference on Harmonisation Good Clinical Practice Guidelines and the Declaration of Helsinki. The study protocols and all documentation were approved by the Institutional Review Boards or Independent Ethics Committees at each investigational site. All study procedures complied with current Health Insurance Portability and Accountability Act of 1996 (HIPAA) regulations. All recruitment locations were approved by a central institutional review board (E&I IRB #2 – IRB00007807), and all recruitment procedures adhered to the IRB-approved study protocol. All patients provided written informed consent.

## Results

### Qualitative FACIT-Fatigue study

In total, 12 interviews were conducted in February 2017 at two clinical sites (Florida, *n* = 7; Pennsylvania, *n* = 5). The mean age (standard deviation; SD [range]) of patients was 53 (14 [27–80]) years, 6 (50%) patients were male, and 11 (92%) were white. The mean time since diagnosis of PsA (SD [range]) was approximately 10 (9 [1–29]) years. Most patients (*n* = 10, 83%) were currently taking medication/treatment for PsA, including methotrexate (*n* = 5, 42%), adalimumab, etanercept, secukinumab (each *n* = 2, 17%), and others (*n* = 3, 30%).

#### PsA symptoms, concept elicitation

As part of the concept elicitation portion of the interview (part 1; Additional file 2: Appendix 2b), patients were asked to describe their PsA signs and symptoms, rate the severity of their condition, and then rank the importance of their symptoms. Patient-rated severity was based on their symptom experience and the impact on their functioning and well-being.

PsA severity was highly variable, described by patients as mild (*n* = 3, 25%), mild to moderate (*n* = 1, 7%), moderate (*n* = 3, 25%), moderate to severe (*n* = 2, 17%), sometimes moderate and sometimes severe (*n* = 1, 7%), severe at first but diminished (*n* = 1, 7%), or severe (*n* = 1, 8%). PsA signs/symptoms experienced over the past 7 days were fatigue (*n* = 12, 100%), pain (in joints, tendons, or entheses; *n* = 11, 92%), skin-related symptoms (itch, dryness, scaling, redness, bleeding, inflammation, or painful skin; *n* = 9, 75%), joint stiffness (any part of body; *n* = 7, 58%), dactylitis (swelling of entire fingers or toes; *n* = 6, 50%), swelling in other parts of body (*n* = 4, 33%), and other symptoms (*n* = 7, 58%). Seven patients (58%) decisively attributed fatigue directly to PsA. Saturation of PsA signs and symptoms was reached (i.e., no new concepts reported) after completion of the eighth interview.

Additionally, patients ranked each symptom relative to their other symptoms from 0 to 4 (0 is “not important at all”; 4 is “extremely important”). Symptoms rated as “important” or “extremely important” are presented in Table 1.

**Table 1:** Ranking of PsA symptoms by patients in the qualitative study (*N* = 12)

Symptom	Rated as “important” or “extremely important”, n (%)
Pain (in joints, tendons, or entheses)	10 (83)
Fatigue (tired/listless/lack of energy/washed out/low energy/weak)	7 (58)
Dactylitis (swelling of entire finger[s] or toe[s])	6 (50)
Swelling (not in finger[s] or toe[s])^a^	5 (42)
Skin-related symptoms (itch, dryness, scaling, redness, bleeding, inflammation, painful skin)	5 (42)
Joint stiffness (any part of the body)	3 (25)
Other symptoms^b^	6 (50)

^a^Swelling described as visible inflammation in the feet/ankles (*n* = 4), hips (*n* = 2), jaw (*n* = 1), elbows (*n* = 1), or knees (*n* = 1)

^b^Other symptoms that were ranked as important or extremely important include feeling depressed/down (*n* = 2), burning in knees (*n* = 1), cramping in the knees (*n* = 1), thumb “twisting” (*n* = 1), and weak hands (*n* = 1)

*PsA* psoriatic arthritis

#### FACIT-Fatigue cognitive debriefing

Subsequently to the concept elicitation portion of the interview, the debriefing portion of the interview (part 2) focused on asking patients to complete the FACIT-Fatigue questionnaire and to provide feedback.

Mean total FACIT-Fatigue score (SD [range]) was 27.1 (10.8 [13–44]) out of a possible maximum score of 52, with this low value, relative to the total score, indicating higher fatigue. Mean Experience domain (SD [range]) score was 7.4 (4.4 [1–15]; highest possible score 20), and average Impact domain score (SD [range]) was 19.7 (6.8 [12–29]; highest possible score 32). During the FACIT-Fatigue interview, patients with PsA generally provided positive feedback on the instrument. All 12 patients commented that completing the questionnaire was “quick,” “easy,” “straightforward,” and “fine”, and found the instructions, item wording and response options clear and easily understood. Overall impressions of the items were favorable, although one patient indicated that the first four items were repetitive (fatigued, weak all over, listless [washed out], tired).

The recall period (past 7 days) was correctly understood by most patients (*n* = 7, 58%); however, other patients (*n* = 5, 42%) did not use the correct recall period, instead reporting their fatigue experiences over the “past month”, “in general”, “today”, “yesterday”, “all the time”, and “during the day”. Two of these patients reported that they read the instructions but decided to consider a different recall period for their answers. Most patients considered FACIT-Fatigue items 1–9 and 12 (range *n* = 10 [83%] to *n* = 12 [100%]) to be relevant to their experience with PsA. Items 11 “I need help doing my usual activities” and 13 “I have to limit my social activity because I am tired” were considered relevant by 9 patients each (75%). Item 10 “I am too tired to eat” was not considered relevant by 8 patients (67%).

Most patients (*n* = 9, 75%) reported that there were no important fatigue-related concepts missing from the questionnaire. The remaining three patients (25%) provided suggestions for improvements to existing items, and for additional items/concepts, including making a distinction between physical and mental fatigue (*n* = 2) and asking patients how they relieve their fatigue. One patient suggested incorporating questions that addressed the mental and emotional aspect of PsA.

Based on the current findings, no changes to the FACIT-Fatigue items and response options were recommended. However, given that more than half of patients did not find item 10 to be relevant to them personally, further exploration of this item in an additional PsA population is recommended. Additionally, given that a sizeable number of patients did not focus on the correct recall period, it may be useful to further highlight the recall period when using the instrument (e.g., emboldening or underlining).

### Psychometric analysis of FACIT-Fatigue in PsA

#### Confirmatory analysis model

The FACIT-Fatigue measurement model was tested using confirmatory factor analysis, which included two first-order factors (representing Experience and Impact domains) and one aggregated second-order factor (representing total score). CFI indices were 0.92 and 0.93 for PD1 and PD2, respectively, and standardized factor loadings were > 0.4 for all items. Supplemental analyses using bifactor modeling supported this, with CFI indices of 0.96 and 0.97 for PD1 and PD2, respectively.

#### Internal consistency reliability

Cronbach’s Coefficient α was ≥0.90 for the FACIT-Fatigue total score, Impact domain, and Experience domain for both PD1 and PD2 (Table 2). All corrected item-to-total correlations were > 0.40 (range 0.42–0.89).

**Table 2:** Internal consistency reliability of FACIT-Fatigue in patients with PsA

	Cronbach’s Coefficient α	Corrected item-to-total correlations, range
PD1: OPAL Beyond baseline data pooled with OPAL Broaden Month 12		
FACIT-Fatigue total score (*N* = 760)	0.95	0.46–0.89
FACIT-Fatigue Experience domain (*N* = 763)	0.93	0.59–0.89
FACIT-Fatigue Impact domain (*N* = 762)	0.91	0.44–0.85
PD2: OPAL Broaden baseline data pooled with OPAL Beyond Month 6		
FACIT-Fatigue total score (*N* = 766)	0.94	0.44–0.89
FACIT-Fatigue Experience domain (*N* = 768)	0.91	0.53–0.86
FACIT-Fatigue Impact domain (*N* = 768)	0.90	0.42–0.83

*FACIT-Fatigue* Functional Assessment of Chronic Illness Therapy-Fatigue,* PD1/2* Pooled Data 1/2, *PsA* psoriatic arthritis

#### Test-retest reliability'

An acceptable test-retest reliability was observed for FACIT-Fatigue Experience domain (ICC = 0.80), Impact domain (0.83), and total score (0.83) using pooled data from the OPAL Broaden and OPAL Beyond RCTs. Test-retest reliability assessments for each separate RCT were also acceptable (Additional file 3: Appendix 3, Table S1).

#### Convergent validity

The correlation between the FACIT-Fatigue domains and other scales used in phase III RCTs was estimated using PD1 and PD2. With the exception of the Health Transition Item (which has a recall period of 1 year), correlations between FACIT-Fatigue and SF-36 domains generally exceeded 0.60 (all were > 0.50; *P* < 0.0001; Table 3). The correlation between FACIT-Fatigue total score and Experience domain and SF-36 Vitality domain was > 0.80 (*P* < 0.0001). FACIT-Fatigue domain scores also correlated with ISI, DLQI total score, PtGA, PtSA, and PtJA (correlations > 0.4).

**Table 3:** Correlations to assess the convergent validity of FACIT-Fatigue vs. other measures in patients with PsA

	PD1: OPAL Beyond baseline data pooled with OPAL Broaden Month 12 data, Pearson correlation coefficient			PD2: OPAL Broaden baseline data pooled with OPAL Beyond Month 6 data, Pearson correlation coefficient		
FACIT-Fatigue vs.:	Experience domain	Impact domain	Total score	Experience domain	Impact domain	Total score
SF-36						
Bodily pain	0.71	0.69	0.72	0.66	0.64	0.68
General health	0.57	0.55	0.58	0.59	0.55	0.59
Mental health	0.65	0.64	0.66	0.65	0.65	0.67
Physical functioning	0.64	0.69	0.69	0.56	0.62	0.62
Role emotional	0.63	0.70	0.70	0.56	0.64	0.63
Role physical	0.71	0.75	0.76	0.63	0.71	0.70
Social functioning	0.71	0.78	0.78	0.66	0.73	0.72
Vitality	0.83	0.75	0.81	0.81	0.75	0.81
Health transition	−0.33	−0.29	−0.32	−0.35	−0.33	−0.35
Mental component	0.68	0.70	0.71	0.66	0.69	0.70
Physical component	0.64	0.65	0.67	0.58	0.60	0.61
ISI	−0.42	−0.37	−0.41	−0.43	−0.41	−0.44
DLQI total score	−0.43	−0.43	−0.44	−0.43	−0.48	−0.48
PtGA	−0.64	−0.60	−0.64	−0.61	−0.57	−0.61
PtJA	−0.65	−0.62	−0.65	−0.61	−0.57	−0.61
PtSA	−0.47	−0.45	−0.47	−0.44	−0.44	−0.46

All results *P* < 0.0001. Correlation coefficients are all in the expected direction

*DLQI* Dermatology Life Quality Index, *FACIT-Fatigue* Functional Assessment of Chronic Illness Therapy-Fatigue, *ISI* Itch Severity Item; *PD1/2* Pooled Data 1/2, *PtGA* Patient’s Global Assessment of Psoriasis and Arthritis (a component of the PtGJS-VAS), *PtGJS* Patient’s Global Joint and Skin Assessment, *PtJA* Patient’s Joint Assessment (a component of the PtGJS-VAS), *PtSA* Patient’s Skin Assessment (a component of the PtGJS-VAS), *PsA* psoriatic arthritis, *SF-36* Short Form Survey-36, *VAS* Visual Analog Scale

#### Defining the clinically important difference for FACIT-Fatigue domains

CID for FACIT-Fatigue was defined by employing a longitudinal RMM to estimate the relationship between PtGA score and FACIT-Fatigue domains, and linked to a 17 mm change (one category difference on a 7-point scale) on the PtGA. Pooled data showed that PtGA had a substantial correlation with FACIT-Fatigue domains at all time points (with values between 0.5 and 0.7 for post-treatment time points) and with correlations < 0.5 at baseline.

The CID for the FACIT-Fatigue total score was 3.1, and for FACIT-Fatigue Experience and Impact domains was estimated to be 1.5 and 1.7, respectively (Table 4). In the sensitivity analysis, CIDs for each RCT were similar.

**Table 4:** Clinically important difference (CID) and responder definition (RD) estimations for FACIT-Fatigue in patients with PsA

Model	Analysis	Anchor	Study data	Experience domain	Impact domain	Total score
				Clinically important difference (SE) [95% CI]		
Repeated measures model-CID	Main analysis	PtGA	Pooled	1.45 (0.04) [1.38, 1.52]	1.73 (0.05) [1.63, 1.82]	3.12 (0.08) [2.97, 3.27]
	Sensitivity analysis	PtGA	OPAL Broaden	1.39 (0.05) [1.30, 1.48]	1.66 (0.06) [1.53, 1.78]	3.00 (0.10) [2.80, 3.19]
			OPAL Beyond	1.53 (0.06) [1.42, 1.64]	1.82 (0.08) [1.67, 1.97]	3.29 (0.12) [3.04, 3.53]
		SF-36 Vitality	Pooled	1.06 (0.01) [1.03, 1.08]	1.21 (0.02) [1.17, 1.25]	2.25 (0.03) [2.18, 2.31]
				Effect size (SD)		
Repeated measures model-CID	Main analysis	PtGA	Pooled	8.73 (4.54)	18.93 (6.88)	27.66 (10.95)
	Sensitivity analysis	PtGA	OPAL Broaden	9.13 (4.34)	19.56 (6.57)	28.69 (10.47)
			OPAL Beyond	8.30 (4.72)	18.25 (7.14)	26.54 (11.35)
				Responder definition (SE) [95% CI]		
Repeated measures model-RD	Main analysis	SGIC	Pooled	1.67 (0.09) [1.49, 1.86]	2.12 (0.12) [1.88, 2.37]	3.76 (0.20) [3.37, 4.15]
	Sensitivity analysis		OPAL Broaden	1.54 (0.12) [1.31, 1.77]	2.02 (0.15) [1.72, 2.33]	3.52 (0.25) [3.03, 4.01]
			OPAL Beyond	1.91 (0.16) [1.60, 2.23]	2.30 (0.20) [1.90, 2.70]	4.19 (0.33) [3.54, 4.85]

Pooled data: OPAL Broaden and OPAL Beyond

*CI* confidence interval, *CID* clinically important difference, *FACIT-Fatigue* Functional Assessment of Chronic Illness Therapy-Fatigue, *PsA* psoriatic arthritis, *PtGA* Patient’s Global Assessment of Psoriasis and Arthritis, *RD* responder definition, *RMM* repeated measures model, *SD* standard deviation, *SE* standard error, *SF-36* Short Form Survey-36, *SGIC* Subject Global Impression of Change

#### Estimation of the responder definition for FACIT-Fatigue domains

An RMM was applied to estimate RD and examine the relationship between FACIT-Fatigue domains and SGIC score as the anchor (see Additional file 2: Appendix 2a). SGIC is based on PtGA change from baseline, but has only 3 categories: “worse” (change from baseline ≥10 mm; value of − 1), “the same” (change from baseline < 10 mm; value of 0), and “better” (change from baseline ≤ − 10 mm; value of + 1).

RD for the FACIT-Fatigue total score was 3.8, and estimated to be 1.7 and 2.1 for FACIT-Fatigue Experience and Impact domains, respectively. In the sensitivity analysis, RDs for the individual RCTs were similar (Table 4). Since a whole number would need to be assigned to denote improvement in an individual, this would therefore appear as 4 points for the FACIT-Fatigue total score, and 2 points for each of the domain scores.

#### Known-groups validity

The known-groups validity analysis was based on a RMM-CID model and evaluated by analyzing the differences in mean FACIT-Fatigue domain scores between the “remission/low disease activity group” and the “active disease group”, (PtGA score of 0 mm, i.e., “excellent”) and the “active disease group” (PtGA score of 100 mm, i.e., “poor”). Differences in the FACIT-Fatigue domain scores and total score between “remission/low disease activity group” and the “active disease group” were statistically different; effect sizes of all differences considered large (all > 1.4), constituting a significant and considerable difference between the groups (Table 5).

**Table 5:** Known-groups validity for FACIT-Fatigue in patients with PsA

Analysis	Differences in the FACIT-Fatigue domain scores between the “remission/low disease activity group” and the “active disease group”					
	Experience domain		Impact domain		Total score	
	Difference (95% CI)	Effect size	Difference (95% CI)	Effect size	Difference (95% CI)	Effect size
Anchor as a continuous variable	8.52 (8.11, 8.93)	1.88	10.15 (9.59, 10.71)	1.48	18.35 (17.46, 19.24)	1.68
Anchor as a categorical variable	8.22 (7.52, 8.91)	1.81	10.06 (9.12, 10.99)	1.46	17.89 (16.40, 19.38)	1.63

Pooled data: OPAL Broaden and OPAL Beyond

*CI* confidence interval, *FACIT-Fatigue* Functional Assessment of Chronic Illness Therapy-Fatigue, *PsA* psoriatic arthritis

#### Ability to detect change

**Fig. 1:**
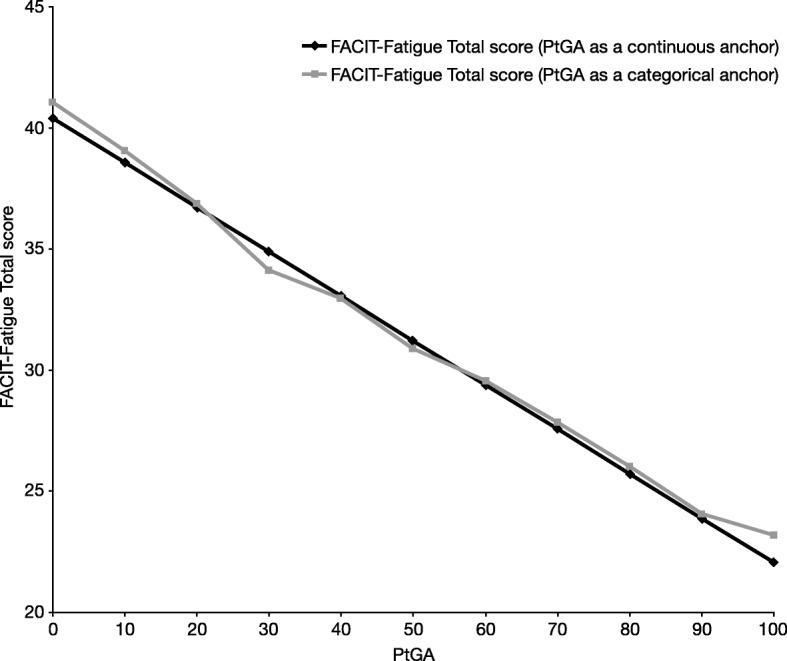
Relationship between FACIT-Fatigue total score and PtGA as a continuous or categorical anchor. *FACIT-Fatigue*: Functional Assessment of Chronic Illness Therapy-Fatigue; *PtGA*: Patient’s Global Assessment of Psoriasis and Arthritis

The ability to detect change analysis was based on a RMM-CID model. Figure 1 compares changes in FACIT-Fatigue total scores with changes in the PtGA scores, and indicates that a patient’s state (as measured by FACIT-Fatigue) changes with respect to the PtGA.

## Discussion

Fatigue is recommended as a core domain to measure in RCTs evaluating treatment effects for psoriatic arthritis [4]. This study evaluated the content validity and quantitative measurement properties to assess whether FACIT-Fatigue is fit for purpose as a measure to evaluate this important domain in RCTs in patients with PsA. The US Food and Drug Administration (FDA) patient-reported outcome (PRO) guidance adds that for labeling claims, adequate evidence is required to support the content validity, construct validity, reliability, and ability of the measure to detect change in the target population of interest [9]. This mixed-methods study evaluated these qualitative and quantitative measurement properties of the FACIT-Fatigue in patients with PsA.

The majority of patients reported experiencing fatigue that was directly attributed to their PsA condition. This confirms the importance of fatigue symptoms in patients with PsA and is consistent with other studies that identify improvements in fatigue as a key outcome signifying improvement in their condition [4, 34, 35]. Furthermore, the reliability of reporting the physical and mental concepts of FACIT-Fatigue (Impact and Experience domains) is also consistent with the reliability of these concepts in other patients with other conditions, such as spinal cord injuries [36].

The cognitive interview allowed for the conclusion that patients provided overall positive feedback on the FACIT-Fatigue questionnaire, finding it to be comprehensive and relevant to their experience of fatigue with PsA. Results were similar to a study in patients with RA, where 15 of 17 patients stated that FACIT-Fatigue items were relevant to them [18]. Notably, item 10 “I am too tired to eat” was considered the least relevant item in both this study (8/12 patients, 67%) and the study in RA (9/17 patients, 53%) [18]. In this study, the instructions, item concepts, and response options were well-understood by most patients. Most correctly understood the recall period; however, some did not use the correct recall period. Overall, no changes to the FACIT-Fatigue items and response options were recommended, although in future studies it may be worthwhile testing item 10 further, and also emboldening or underlining the recall period for added generalizability and accuracy.

In the psychometric analysis of RCT data in patients with PsA, the second-order confirmatory factor analysis model supported the measurement model of the FACIT-Fatigue scale as an overall score with two distinguishable domains (“Experience” and “Impact”) in addition to a global domain (overall score). Supplemental bifactor confirmatory factor analysis also supported this measurement structure. Good internal consistency reliability was seen in FACIT-Fatigue; Cronbach’s Coefficient α’s were ≥ 0.90, and all corrected item-to-total correlations were > 0.4. The ability to detect change, while part of instrument validity [37], is of sufficient importance to PRO measurement in longitudinal studies that it may be analyzed separately [29, 38], as done here. These findings demonstrated the sensitivity of FACIT-Fatigue to changes in PtGA scores. Results provided evidence that FACIT-Fatigue is equally sensitive to increases and decreases in PtGA scores, showing that when a patient’s experience of fatigue is predicted to change (i.e., change in severity of illness measured by PtGA), the values for FACIT-Fatigue also change. The test-retest reliability analysis observed an acceptable ICC (≥ 0.80) for all FACIT-Fatigue domains.

FACIT-Fatigue Impact and Experience domains were observed to correlate with almost all measured outcomes, suggesting that the physical and mental impacts of fatigue are closely linked to patient perception of PsA. Furthermore, FACIT-Fatigue total score was observed to correlate strongly (*r* > 0.80) with the SF-36 Vitality domain. As both fatigue and dermatological symptoms improve with PsA therapies (e.g., etanercept or adalimumab) [39, 40], it was expected here that FACIT-Fatigue scores would correlate with dermatological scores. However, ISI, DLQI, and PtSA scores (− 0.37 to − 0.48) were numerically lower than the correlations of FACIT-Fatigue scores with PtJA scores (− 0.57 to − 0.65), potentially indicating that FACIT-Fatigue is more related and sensitive to the effects of arthritis than psoriasis.

Different terms and approaches have been used to characterize and formulate a CID (between-group difference) and RD (within-individual or within-group change) for PROs [41, 42], and some have been used in rheumatology [43, 44]. Here, the CID of FACIT-Fatigue is the clinically relevant difference in scores between two treatment groups, and the RD is the amount of improvement an individual patient would have to report to indicate experience of a relevant treatment benefit. It is therefore akin to a CID that has been reported in rheumatology [43, 44]. RD was estimated using a RMM, based on the algorithm recommended in the FDA guidance [9].

FACIT-Fatigue domain scores were significantly different between the “remission/low disease activity group” and the “active disease group”, corroborating known-groups validity. The CID was defined using PtGA as an anchor and for the FACIT-Fatigue total score was 3.1. This is consistent with the value of 3–4 points reported in patients with other diseases, including cancer and RA [12, 45]. The RD for the FACIT-Fatigue total score was estimated to be a 4-point improvement, based on the average 3.8-point improvement associated with SGIC improvement. Overall, results were highly consistent with previous findings for FACIT-Fatigue [12, 15].

The 13 items of FACIT-Fatigue are also embedded in the Patient-Reported Outcomes Measurement Information System^®^ (PROMIS^®^) Fatigue item bank, a 95-item fatigue assessment tool. This can be used as either a computerized adaptive test or a fixed-length short form, and was designed to compare differences across a range of chronic conditions, enabling comparative effectiveness research [46]. The use of fatigue short forms from PROMIS has been validated in RA [47], and the current research provides strong evidence supporting the validity of the FACIT-Fatigue scale and its measurement properties in patients with PsA, which opens up the possibility for including PsA data in the unifying PROMIS metric.

Advantages/strengths of this study included the self-reported nature of the PRO measures, and the systematic collection of clinical and PRO data. Moreover, patients’ demographic and disease characteristics were well balanced. However, as data were taken from RCTs with specific eligibility criteria, generalizing these data to real-world populations may not be possible. Test-retest reliability, performed separately for OPAL Broaden and OPAL Beyond, confirmed the acceptability of the test-retest reliability from the pooled results.

Limitations of these analyses include that estimated CID (between-group difference) and RD (within-individual or within-group change) may vary due to different methodology and natural sampling variation, along with other considerations, and may not necessarily represent a minimal value [41]. Additionally, there is no current consensus in the literature as to what may constitute a meaningful change. As such, while distribution-based methods were used in this study, it must be noted that individual-based methods may also be used to define a meaningful change.

A further limitation may include changes in the anchor measures not fully reflecting CID in FACIT-Fatigue. Moreover, it would have been desirable to perform test-retest reliability assessments before treatment (i.e., during the screening [test] visit, and baseline [retest] visit); however, as these assessments were not available, test-retest reliability was performed in a stable group of patients at baseline and Month 1 (based on a < 10 mm difference in PtGA from baseline to Month 1), and provided the largest number of patients within the shortest possible time period.

It should be noted that in the qualitative interviews, the reported range of scores (range 13–44) did not include those for the most severe fatigue; therefore, concepts considered not relevant (e.g., “I’m too tired to eat”) may remain relevant in patients with more severe fatigue. It also remains unclear how specific the patient feedback reported in this study is to the FACIT-Fatigue measure, or if this is also applicable to similar measures (e.g., Multidimensional Assessment of Fatigue). Furthermore, use of pooled data from two RCTs with different eligibility criteria, and use of different time points from each study, may confound the results.

## Conclusion

In summary, the findings of this study, including analyses performed for the first time using data from RCTs in PsA, suggest that the content of the FACIT-Fatigue scale is valid for use as an endpoint to measure fatigue in PsA RCTs. Qualitative interviews identified the concepts relevant and important to patients, and demonstrated that there were no fatigue-related concepts missing from the FACIT-Fatigue scale. The FACIT-Fatigue items and response options were also found to not require any changes. However, further testing of item 10 (“I am too tired to eat”) may be advantageous to ensure that this item is relevant to a more general population.

Analysis of FACIT-Fatigue data from two PsA RCTs showed good content validity and reliability, and a strong correlation with other disease measures. These conclusions, in conjunction with confirmations of CID and RD consistent with previous findings, support the use of FACIT-Fatigue in PsA RCTs.

## Additional file

Additional file 1: Appendix 1: The FACIT-Fatigue scale. (DOCX 255 kb)

Additional file 2: Appendix 2: Patients and methods [19, 21]. (DOCX 870 kb)

Additional file 3: Appendix 3: Test/retest reliability of FACIT-Fatigue in patients with PsA. (DOCX 12.4 kb)

## Abbreviations

BID: Twice daily
CASPAR: ClASsification criteria for Psoriatic Arthritis
CFI: Comparative Fit Index
CI: Confidence interval
CID: Clinically important difference
csDMARD: Conventional synthetic disease-modifying antirheumatic drug
DLQI: Dermatology Life Quality Index
FACIT-Fatigue: Functional Assessment of Chronic Illness Therapy-Fatigue
FDA: US Food and Drug Administration
HIPAA: Health Insurance Portability and Accountability Act of 1996
ICC: Intraclass Correlation Coefficients
IR: Inadequate response
IRB: Institutional review board
ISI: Itch Severity Item
PD: Pooled data
PRO: Patient-reported outcome
PROMIS^®^: Patient-Reported Outcomes Measurement Information System^®^
PsA: Psoriatic arthritis
PtGA: Patient’s Global Assessment of Psoriasis and Arthritis
PtGJS-VAS: Patient’s Global Joint and Skin Assessment – Visual Analog Scale
PtJA: Patient’s Joint Assessment
PtSA: Patient’s Skin Assessment
RCT: Randomized controlled trial
RD: Responder definition
SD: Standard deviation
SF-36: Short Form Survey-36
SGIC: Subject Global Impression of Change
TNFi: Tumor necrosis factor inhibitor
