# Supplementary material for: Content validity and psychometric evaluation of Functional Assessment of Chronic Illness Therapy-Fatigue in patients with psoriatic arthritis
Source: J Patient Rep Outcomes. 2019 May 20;3:30. doi: 10.1186/s41687-019-0115-4 (PMC6527714; doi:10.1186/s41687-019-0115-4)
Supplement: Supplementary file 1 — Appendix 1: The FACIT-Fatigue scale. (DOCX 255 kb) [file 41687_2019_115_MOESM1_ESM.docx]

ADDITIONAL FILE 1

APPENDIX 1

## Figure S1 The FACIT-Fatigue scale


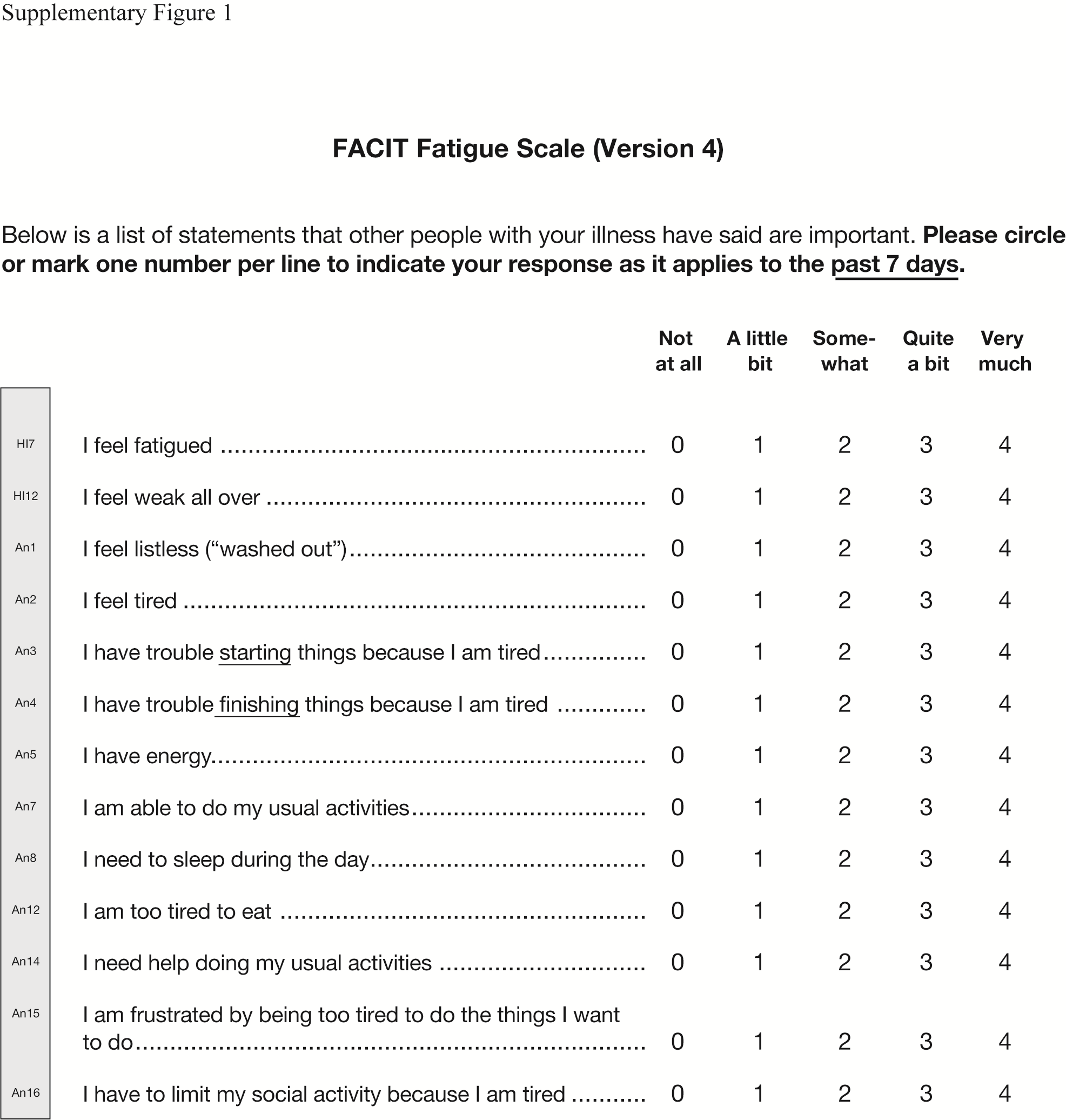


The FACIT-Fatigue scale is owned and copyrighted by, and the intellectual property of, David Cella, PhD. Reproduced with permission from David Cella, PhD
FACIT-Fatigue: Functional Assessment of Chronic Illness Therapy-Fatigue
