# Supplementary material for: Content validity and psychometric evaluation of Functional Assessment of Chronic Illness Therapy-Fatigue in patients with psoriatic arthritis
Source: J Patient Rep Outcomes. 2019 May 20;3:30. doi: 10.1186/s41687-019-0115-4 (PMC6527714; doi:10.1186/s41687-019-0115-4)
Supplement: Supplementary file 2 — Appendix 2: Patients and methods [19, 21]. (DOCX 870 kb) [file 41687_2019_115_MOESM2_ESM.docx]

ADDITIONAL FILE 2

APPENDIX 2A

# Patients and methods

## Qualitative FACIT-Fatigue study

### Inclusion/exclusion criteria

Diagnosis of psoriatic arthritis (PsA) was based on ClASsification criteria for Psoriatic ARthritis (CASPAR) criteria [46] for ≥ 6 months prior to screening (clinician-reported); active PsA was defined as having clinically apparent psoriasis (skin or nails) and active inflammatory synovitis involving at least one peripheral joint (i.e., one tender/painful and one swollen joint). Exclusion criteria included: pregnancy at time of screening, or 6 months prior; participation in an interventional medical research study or RCT at the time of screening or within 4 weeks prior; pre-existing medical conditions that may have confounded PsA reporting; current (at time of screening) or recent history of uncontrolled renal, hepatic, hematological (including anemia), gastrointestinal, metabolic (including hypercholesterolemia), endocrine, pulmonary, cardiovascular, or neurologic disease; history of alcohol or drug abuse unless in full remission for greater than 6 months prior to interview; and confirmed cognitive impairment or patient otherwise judged to be unable to participate by the study Principal Investigator(s).

***Analysis of qualitative data***

Qualitative data was analyzed using ATLAS.ti qualitative data analysis software version 7.5.15 [20], using methodology described previously [21]. ATLAS.ti software was designed for the qualitative analysis of textual, graphical, audio, and video data. ATLAS.ti is fundamentally a concept database that allows the researcher to create and enter names of concepts, or "codes," to be used for conceptualizing large amounts of qualitative data. The program allows the analyst to organize and relate these concepts to each other in order to evaluate the underlying structure of the qualitative data. Using ATLAS.ti, qualitative data can be systematically analyzed, coded, and compared.

Firstly, all interviews were digitally recorded, and audio recordings were transcribed. An Evidera project team member reviewed the interview transcripts for content and removed any participant‑identifying information. The final transcript was labeled as "clean," and this final version was analyzed. The analysis of the cleaned transcripts involved developing a coding dictionary based on the structure of the interview guide (Additional File 2: Appendix 2b). The coding dictionary was developed by Evidera staff trained in identifying key concepts that were translated into codes that were useful for summarizing the results. This was then uploaded in ATLAS.ti in order to begin analysis.

Secondly, two scientific staff members independently coded the first several transcripts and their coding was compared for consistency. A third scientific staff member reviewed and provided feedback on the coding and reviewed subsequent coding as needed until this was consistent between coders. An iterative coding approach was applied moving between consecutive transcripts and new codes that emerged, based on previously described methods (constant comparative method) [23]. The coders attached relevant "codes" to concepts mentioned within each transcript.

Participant quotes were grouped and summarized by thematic code, and coding outputs were generated based on each utilized code. The utilized codes were entered into a saturation grid, where concepts identified in each interview were analyzed with the goal of comparing and tallying the amount of novel information that is observed in each subsequent interview. Saturation is defined as the point at which no substantially new themes, descriptions of a concept, or terms are introduced as additional interviews are conducted. The number of patients needed to reach saturation is largely driven by the complexity of a concept and the diversity of the population of patients (e.g., age, severity) who share the commonality of interest (disease, treatment, or other relevant health-related experience). Although larger sample sizes (i.e., additional interviews) can contribute to confidence in content validity, saturation is the point at which additional sampling offers no new information and serves no purpose [24, 47].

## Psychometric analysis of FACIT-Fatigue in PsA

### Defining the clinically important difference for FACIT-Fatigue domains

The CID refers to the difference in scores between two treatment groups that can be considered clinically relevant. CID was estimated using a RMM to compare the relationship between the PtGA score and domains of FACIT-Fatigue in PD3. A domain (Impact or Experience) of FACIT-Fatigue (including total score) is the outcome and PtGA is a continuous anchor or categorical anchor.

When using PtGA as an anchor, it is important to note that it is a VAS; hence, there are no clear patient-selected categories to use as a basis to define a CID. For example, for this study a single-item instrument such as Patient Global Impression-Severity would have used a 7‑point scale as follows: Please rate the severity of your PSORIASIS and ARTHRITIS: 1‑“not present,” 2-“very mild,” 3-“mild,” 4-“ moderate,” 5-“moderately severe,” 6-“severe,” 7-“extremely severe”. If it is assumed that 100 mm VAS PtGA (used in OPAL Broaden and OPAL Beyond) can be linearly approximated by a 7-point scale, then it can be assumed that a value of 17 mm could be representative of the one-category difference and could be used to estimate the CID for a FACIT-Fatigue domain [30, 31]. For instance, a difference in FACIT-Fatigue domain that corresponds to a 17 mm difference on PtGA represents estimation of the CID (note that 17 mm = 100 mm/6, where 6 is the number of pairwise adjacent categories).

Another important aspect is the representation of the CID in terms of the effect sizes. The standard deviation for the total score at baseline was 10.95, which means that the value of the 3.12 CID estimation for the total score corresponds to an effect size of 0.28, which is considered a small-to-medium effect size (effect size provides a general set of thresholds or benchmarks through adjectival descriptors on the impact of an intervention, with values of 0.2 generally regarded as “small”, 0.5 as “medium”, and 0.8 as “large”).

Additional analyses using SF-36 Vitality domain as an anchor gave close results for the CID estimations (approximately 30% smaller) and were generally supportive of the main analyses (note: these analyses were based on the assumption that a 3-point change in SF-36 Vitality domain can be mapped into an estimation of the CID for FACIT-Fatigue domains.

### Defining the responder definition for FACIT-Fatigue domains

The responder definition (RD) refers to the amount of change an individual patient would have to report to indicate that a relevant treatment benefit has been experienced. The value of RD was estimated using a RMM to compare the relationship between a new anchor, the “Subject Global Impression of Change” (SGIC) score, and domains of FACIT-Fatigue. SGIC is based on PtGA and was formed using the following algorithm: if PtGA change from baseline is less or equal to minus 10 mm (negative change), this corresponds to “better” (SGIC = 1); if PtGA change from baseline in absolute value is less than 10 mm, this means “the same” (SGIC = 0); if PtGA change from baseline is more or equal to plus 10 mm (positive change), this means “worse” (SGIC = -1). SGIC was treated as a continuous variable, which imposes a linear relationship between change in a FACIT-Fatigue domain and SGIC. In sensitivity analyses SGIC was modeled as a categorical variable.

The value of RD for the total score corresponds to an effect size of 0.34, which is considered a small-to-medium effect size.

**APPENDIX 2B**

# Concept Elicitation & Cognitive Interview Guide (EVA-20175)

**Overview**: The combined concept elicitation and cognitive interview guide is developed to guide the discussion with patients with psoriatic arthritis (PsA). The interviewer will have the flexibility to modify the order or phrasing of questions and/or probes in this guide in order to obtain greater clarification, minimize redundancy if relevant concepts emerge earlier than expected, and to further explore interesting or novel concepts. However, it is considered *good practice* to stick to the guide question wording as best as possible, where it makes sense. Additionally, please encourage a **think aloud** approach, before probing on any of the items and ensure you are getting the patient’s own words as much as possible

Blue= For Interviewer only

Black= Read to participant

**Interview Structure:**

1. **Ask participants to turn off their cell phones.**
2. Explain the study objectives and describe what will occur during the interview with the participant.
3. Review the IRB-approved informed consent form with the participant, and provide an opportunity for the participant to ask questions.
4. Obtain written informed consent from the participant. Consent will be obtained prior to the start of any study procedures or the conduct of the interview.
5. Start the audio recording and begin interview.
6. Conduct Concept Elicitation portion of the interview.
7. Administer the FACIT-F questionnaire. Check that all items are complete being moving on.
8. Conduct the Cognitive Interview portion of the interview.
9. End interview and recording, provide sociodemographic form for participants to complete. Check all field are complete before moving on.
10. Thank the participant for their time and remunerate using a ClinCard. The interviewer can set up the ClinCard with the participant’s personal information if they require a pin, or can use dummy information if they do not require a pin.

Important: The interview is expected to take up to 90 minutes (please take a break, if needed by the participant).

**START INTERVIEW: INTRODUCTION (UP TO 10 MIN)**

Good (morning/afternoon/evening). [Introduce self]

Thank you for coming in today. Let me tell you a little about what we are going to be doing here today. First, I am going to ask you a few questions about your experience with psoriatic arthritis, specifically what your symptoms are and how they impact your life. Then, I will ask you to complete a questionnaire, and I will talk with you about the questions to make sure they are easy to understand and answer. As you complete the interview today, I want you to remember that there are no right or wrong answers. You are the expert and are here to share and express your point of view.

**INTERVIEW RULES**

Before we begin, I’m going to cover some things that will make our discussion more productive.

- I may use your first name in the interview, but in later reports no names will be attached to any comments. Your name and any other personally identifiable information will be kept as confidential as possible.
- My role here is to ask questions and to listen. I will also be summarizing information at times. I will ask questions about issues related to your experience, and I will move the discussion from one question to the next to try to keep us on track so that we can finish on time.
- I am not a medical doctor, so I am not qualified to give you any medical advice. I encourage you to follow-up with your regular doctor if you have any questions about your medication or condition after this interview.
- To thank you for your time, we will be paying you $150 in the form of a debit/credit card called a ClinCard following the conclusion of the discussion.
- Lastly, please feel free to let me know if you need a break. You can ask me questions at any time. We will now begin recording. OK?

I would like to have your permission to audio record this interview. This recording will be kept confidential and your name will not be linked with your responses in any way. The recording will be used to help us write a report about the things we talked about here today.

**START HERE**

***Begin Recording*:** This is study EVA-20175 and this is participant ID [insert number here]. Today’s date is [insert date here]. Do I have your permission to record this interview?

- I would like to confirm that you read and signed the consent form? *[****YES****/NO]*
- Also, that we have given you a copy of those forms for your records? *[****YES****/NO]*
- Do you have any questions on the consent form? *[YES/****NO****]*
  - If yes, please ask the participant to elaborate and document the issue while recording.
- **Interviewer should not continue with the interview unless participants reply to the responses in bold (Yes, Yes, No, Yes)**

**CONCEPT ELICITATION OF PSORIATIC ARTHRITIS SIGNS AND SYMPTOMS (UP TO 25 MIN)**

Let’s get started.

1. When/How long ago were you diagnosed with psoriatic arthritis?
2. Would you consider your current psoriatic arthritis mild, moderate or severe?
3. What symptoms have you experienced **over the last 7 days (past week)** due to psoriatic arthritis? Allow participants to spontaneously answer before continuing with probes. If not mentioned, please probe further. Mark below if mentioned any of the signs/symptoms below.

Table 1. Signs/Symptoms of Patient’s PsA

| Sign/ Symptoms Currently Experiencing | Spontaneously mentioned? | Probed? | Importance  (0= Not at all Important to 4= Extremely Important) |
| --- | --- | --- | --- |
| 1. Pain (in joints, tendons, or enthuses- sites where tendons insert to bone) |  |  |  |
| 1. Swelling of entire finger/s or toe/s- (dactylitis) |  |  |  |
| 1. Swelling in other parts of the body |  |  |  |
| 1. Stiffness (any part of the body) |  |  |  |
| 1. Fatigue/ tired/ listless/lack of energy/ washed out/ low energy/ weak/ other ways to describe? |  |  |  |
| 1. Psoriasis – skin related symptoms (itch, dryness, scaling, redness, bleeding, inflammation, painful skin) |  |  |  |
| 1. Other: |  |  |  |

***If he/she does not mention all of these symptoms:***

- Ask if there are any other symptoms that he/she would like to talk about.
- If the answer is ‘no’, then probe on other symptoms too but keep it brief.

***[ONLY if Fatigue was not mentioned to have been experienced over the last 7 days as probed or spontaneous, ask question 4, otherwise skip to question 5]***

1. Have you ever experienced fatigue as a result of your psoriatic arthritis anytime in the past? [YES/NO]
2. So you have experienced [list all symptoms endorsed above] as part of your psoriatic arthritis:

- How important is [symptom x] relative to your other symptoms on a scale from 0 to 4, where 0 is not important at all, 4 is extremely important?
- Repeat this question for all symptoms endorsed in items 3 and 4. (*Document results in Table 1, last column).*

**[ONLY if Fatigue was mentioned to have been experienced by patient as result of their PsA (anytime in past), ask the questions 6-9, otherwise skip to cognitive interview]**

1. How would you describe fatigue to someone who does not have psoriatic arthritis?
2. Does/Did the experience of fatigue impact any of your daily activities? If yes, how?
3. How often do/did you have fatigue?

- *Probe*: Ask about different timeframes – in a day and in a week.

1. When do/did you have fatigue?

- *Probe*: Do/did you have fatigue during the day? At night?
- *Probe*: Are there times when you are more likely to have fatigue?

1. Does/did the severity of fatigue change daily? Weekly? Monthly?

- *Probe*: How would you describe your ‘usual’ severity of fatigue?

**COGNITIVE INTERVIEW: DISCUSSION OF THE FACIT-Fatigue (UP TO 45 MIN)**

Now, we will ask your input on a specific questionnaire. ***Take the FACIT-Fatigue sheet from the participant folder***. Please take as much time as you need to complete this questionnaire as you normally would, say in a doctor’s office.

*Wait for the participant to complete the questionnaire* and mark time it took to complete, with start and finish times*. Check to make sure all items on the FACIT-F have response, if not, clarify with the participant.*

| Time Started: _________________  Time Finished: _________________  Total completion time (in minutes): ________ |
| --- |

**Once the participant completes the questionnaire, ask the following questions:**

1. What is your overall impression of this questionnaire?
2. What did you think about the amount of time it took you to complete the questionnaire?
3. What time period were you thinking about when you answered the questions?
4. Do you think people would be able to remember their fatigue related symptoms over 7 days?
5. You probably noticed that there are some instructions at the top of the page. Can you tell me in your own words what the instructions were asking about?
   1. In your opinion, were the instructions clear? Could the instructions be made clearer?
6. Did you have any difficulty understanding the response options?
7. Do you think these response options were appropriate for these questions?

**ITEM LEVEL QUESTIONS 1-13**

Let’s go through each of the questions. Let’s look at item 1**….** Go through each question in the same format as shown below. Ask about all questions in the FACIT-F.

**ITEM 1: I feel fatigued**

1. Can you tell me in your own words what this item is asking about?
2. Is this item relevant to your own experience with Psoriatic Arthritis?
3. Did you have any difficulty understanding this item?
4. What did you mark as an answer for this item?
   1. Tell me more about why you chose this answer.
   2. What about the other item responses; can you tell me what not at all means; a little bit; somewhat; quite a bit; very much means to you in this context? Be sure to ask about each one separately.
5. If item is unclear or you notice it is unclear the way they described it, please ask (otherwise skip): Would you change this item in any way to make it easier to understand, if anything?
6. Is there anything else you would like to add or mention about this item?

**ITEM 2: I feel weak all over**

1. Can you tell me in your own words what this item is asking about?
2. Is this item relevant to your own experience with Psoriatic Arthritis?
3. Did you have any difficulty understanding this item?
4. What did you mark as an answer for this item?
   1. Tell me more about why you chose this answer.
   2. What about the other item responses; can you tell me what not at all means; a little bit; somewhat; quite a bit; very much means to you in this context? Be sure to ask about each one separately.
5. If item is unclear or you notice it is unclear the way they described it, please ask (otherwise skip): Would you change this item in any way to make it easier to understand, if anything?
6. Is there anything else you would like to add or mention about this item?

**ITEM 3: I feel listless (“washed out”)**

1. Can you tell me in your own words what this item is asking about?
2. Is this item relevant to your own experience with Psoriatic Arthritis?
3. Did you have any difficulty understanding this item?
4. What did you mark as an answer for this item?
   1. Tell me more about why you chose this answer.
   2. What about the other item responses; can you tell me what not at all means; a little bit; somewhat; quite a bit; very much means to you in this context? Be sure to ask about each one separately.
5. If item is unclear or you notice it is unclear the way they described it, please ask (otherwise skip): Would you change this item in any way to make it easier to understand, if anything?
6. Is there anything else you would like to add or mention about this item?

**ITEM 4: I feel tired**

1. Can you tell me in your own words what this item is asking about?
2. Is this item relevant to your own experience with Psoriatic Arthritis?
3. Did you have any difficulty understanding this item?
4. What did you mark as an answer for this item?
   1. Tell me more about why you chose this answer.
   2. What about the other item responses; can you tell me what not at all means; a little bit; somewhat; quite a bit; very much means to you in this context? Be sure to ask about each one separately.
5. If item is unclear or you notice it is unclear the way they described it, please ask (otherwise skip): Would you change this item in any way to make it easier to understand, if anything?
6. Is there anything else you would like to add or mention about this item?

**ITEM 5: I feel trouble *starting* things because I am tired**

1. Can you tell me in your own words what this item is asking about?
2. Is this item relevant to your own experience with Psoriatic Arthritis?
3. Did you have any difficulty understanding this item?
4. What did you mark as an answer for this item?
   1. Tell me more about why you chose this answer.
   2. What about the other item responses; can you tell me what not at all means; a little bit; somewhat; quite a bit; very much means to you in this context? Be sure to ask about each one separately.
5. If item is unclear or you notice it is unclear the way they described it, please ask (otherwise skip): Would you change this item in any way to make it easier to understand, if anything?
6. Is there anything else you would like to add or mention about this item?

**ITEM 6: I have trouble *finishing* things because I am tired**

1. Can you tell me in your own words what this item is asking about?
2. Is this item relevant to your own experience with Psoriatic Arthritis?
3. Did you have any difficulty understanding this item?
4. What did you mark as an answer for this item?
   1. Tell me more about why you chose this answer.
   2. What about the other item responses; can you tell me what not at all means; a little bit; somewhat; quite a bit; very much means to you in this context? Be sure to ask about each one separately.
5. If item is unclear or you notice it is unclear the way they described it, please ask (otherwise skip): Would you change this item in any way to make it easier to understand, if anything?
6. Is there anything else you would like to add or mention about this item?

**ITEM 7: I have energy**

1. Can you tell me in your own words what this item is asking about?
2. Is this item relevant to your own experience with Psoriatic Arthritis?
3. Did you have any difficulty understanding this item?
4. What did you mark as an answer for this item?
   1. Tell me more about why you chose this answer.
   2. What about the other item responses; can you tell me what not at all means; a little bit; somewhat; quite a bit; very much means to you in this context? Be sure to ask about each one separately.
5. If item is unclear or you notice it is unclear the way they described it, please ask (otherwise skip): Would you change this item in any way to make it easier to understand, if anything?
6. Is there anything else you would like to add or mention about this item?

**ITEM 8: I am able to do my usual activities**

1. Can you tell me in your own words what this item is asking about?
2. Is this item relevant to your own experience with Psoriatic Arthritis?
3. Did you have any difficulty understanding this item?
4. What did you mark as an answer for this item?
   1. Tell me more about why you chose this answer.
   2. What about the other item responses; can you tell me what not at all means; a little bit; somewhat; quite a bit; very much means to you in this context? Be sure to ask about each one separately.
5. If item is unclear or you notice it is unclear the way they described it, please ask (otherwise skip): Would you change this item in any way to make it easier to understand, if anything?
6. Is there anything else you would like to add or mention about this item?

**ITEM 9: I need to sleep during the day**

1. Can you tell me in your own words what this item is asking about?
2. Is this item relevant to your own experience with Psoriatic Arthritis?
3. Did you have any difficulty understanding this item?
4. What did you mark as an answer for this item?
   1. Tell me more about why you chose this answer.
   2. What about the other item responses; can you tell me what not at all means; a little bit; somewhat; quite a bit; very much means to you in this context? Be sure to ask about each one separately.
5. If item is unclear or you notice it is unclear the way they described it, please ask (otherwise skip): Would you change this item in any way to make it easier to understand, if anything?
6. Is there anything else you would like to add or mention about this item?

**ITEM 10: I am too tired to eat**

1. Can you tell me in your own words what this item is asking about?
2. Is this item relevant to your own experience with Psoriatic Arthritis?
3. Did you have any difficulty understanding this item?
4. What did you mark as an answer for this item?
   1. Tell me more about why you chose this answer.
   2. What about the other item responses; can you tell me what not at all means; a little bit; somewhat; quite a bit; very much means to you in this context? Be sure to ask about each one separately.
5. If item is unclear or you notice it is unclear the way they described it, please ask (otherwise skip): Would you change this item in any way to make it easier to understand, if anything?
6. Is there anything else you would like to add or mention about this item?

**ITEM 11: I need help doing my usual activities**

1. Can you tell me in your own words what this item is asking about?
2. Is this item relevant to your own experience with Psoriatic Arthritis?
3. Did you have any difficulty understanding this item?
4. What did you mark as an answer for this item?
   1. Tell me more about why you chose this answer.
   2. What about the other item responses; can you tell me what not at all means; a little bit; somewhat; quite a bit; very much means to you in this context? Be sure to ask about each one separately.
5. If item is unclear or you notice it is unclear the way they described it, please ask (otherwise skip): Would you change this item in any way to make it easier to understand, if anything?
6. Is there anything else you would like to add or mention about this item?

**QUESTION 12: I am frustrated by being too tired to do the things I want to do**

1. Can you tell me in your own words what this item is asking about?
2. Is this item relevant to your own experience with Psoriatic Arthritis?
3. Did you have any difficulty understanding this item?
4. What did you mark as an answer for this item?
   1. Tell me more about why you chose this answer.
   2. What about the other item responses; can you tell me what not at all means; a little bit; somewhat; quite a bit; very much means to you in this context? Be sure to ask about each one separately.
5. If item is unclear or you notice it is unclear the way they described it, please ask (otherwise skip): Would you change this item in any way to make it easier to understand, if anything?
6. Is there anything else you would like to add or mention about this item?

**ITEM 13: I have to limit my social activity because I am tired**

1. Can you tell me in your own words what this item is asking about?
2. Is this item relevant to your own experience with Psoriatic Arthritis?
3. Did you have any difficulty understanding this item?
4. What did you mark as an answer for this item?
   1. Tell me more about why you chose this answer.
   2. What about the other item responses; can you tell me what not at all means; a little bit; somewhat; quite a bit; very much means to you in this context? Be sure to ask about each one separately.
5. If item is unclear or you notice it is unclear the way they described it, please ask (otherwise skip): Would you change this item in any way to make it easier to understand, if anything?
6. Is there anything else you would like to add or mention about this item?

**Conclusion of discussion about FACIT-Fatigue (Up to 5 min)**

1. Do you experience fatigue as side effect of any medications you are currently taking?
   - **If yes, please ask:**
     - Do you know what medication may be causing this side effect?
     - How long does fatigue last after taking medication?
     - Is there a difference between fatigue caused by medication and your experience with fatigue as a result of your psoriatic arthritis?
2. Are there any other experiences you have had with fatigue that are not covered in this questionnaire?
3. This questionnaire was developed to specifically measure fatigue related symptoms. How do you think that other people who have psoriatic arthritis, like you do, would react to this questionnaire?
4. If you were developing a questionnaire about fatigue, what would you change from the questionnaire you just completed, if anything?
5. Do you have any other thoughts about the questionnaire that I have not asked about that you would like to share with me?

**WRAP-UP OF INTERVIEW AND COMPLETION OF DEMOGRAPHIC AND OTHER FORMS (UP TO 5 MIN)**

Thank respondents for their time. Request that they complete the Sociodemographic and Clinical form. After completion and full check for completeness, give them the ClinCard and enter their information in the ClinCard system to activate the card.

APPENDIX 2C

## Figure S2 FACIT-Fatigue second-order confirmatory factor analysis model


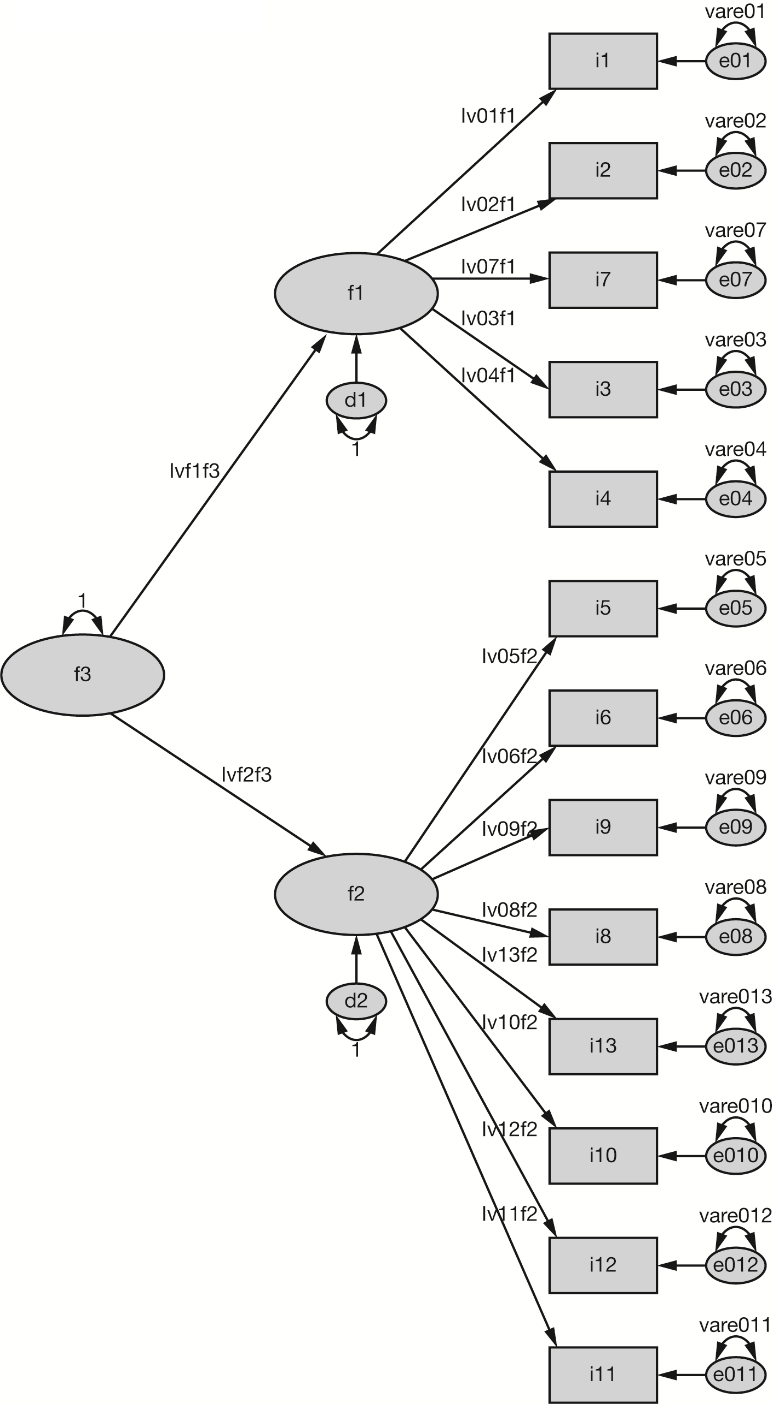


In the confirmatory factor analysis model, the “Experience” and “Impact” domains were represented by latent (unobserved) variables f1 and f2, respectively. The second-order aggregate latent factor f3 subsumes both factors f1 and f2. The factor loadings were represented by “1vf” and “1f” path coefficients (e.g., 1vf3f2 represented by loading factor from f3 to f2, and 1v02f1 represented the factor loading from f1 to item or variable 2). The disturbance terms for the factors were represented by “d” (e.g., d2 represents the disturbance terms associated with factor 2). The error terms for the observed items were represented by “e” (e.g., e1 represents the error term associated with item or variable 1)
FACIT-Fatigue: Functional Assessment of Chronic Illness Therapy-Fatigue

**Figure S3.** Second-order confirmatory factor analysis model factor loadings


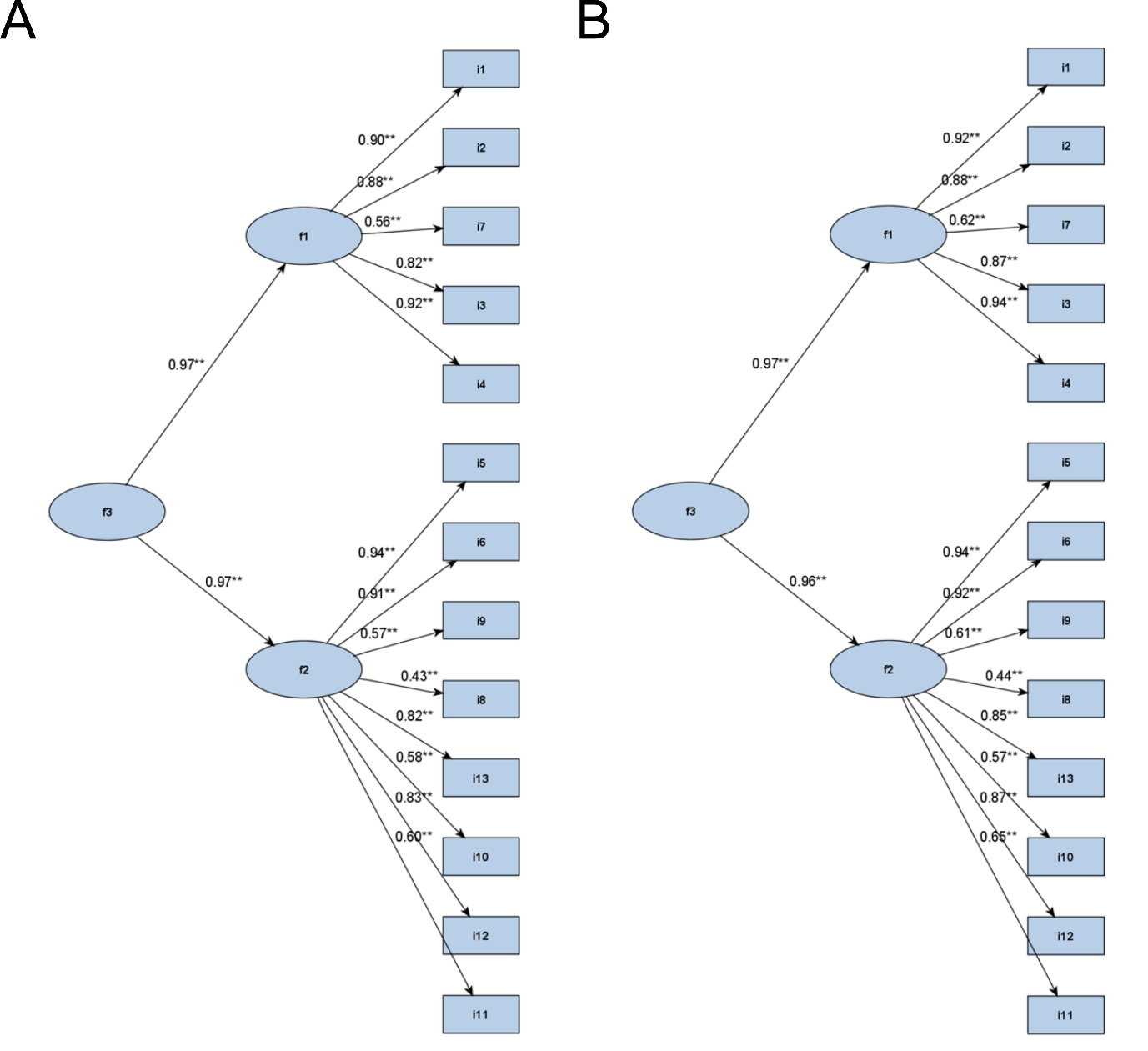


These diagrams show the standardized solution factor loading between A) OPAL Broaden at baseline and OPAL Beyond at Month 6 and B) OPAL Beyond at baseline and OPAL Broaden at Month 12

In the confirmatory factor analysis model, the “Experience” and “Impact” domains were represented by latent (unobserved) variables f1 and f2, respectively. The second-order aggregate latent factor f3 subsumes both factors f1 and f2

**Figure S4.** Bifactor confirmatory factor analysis model


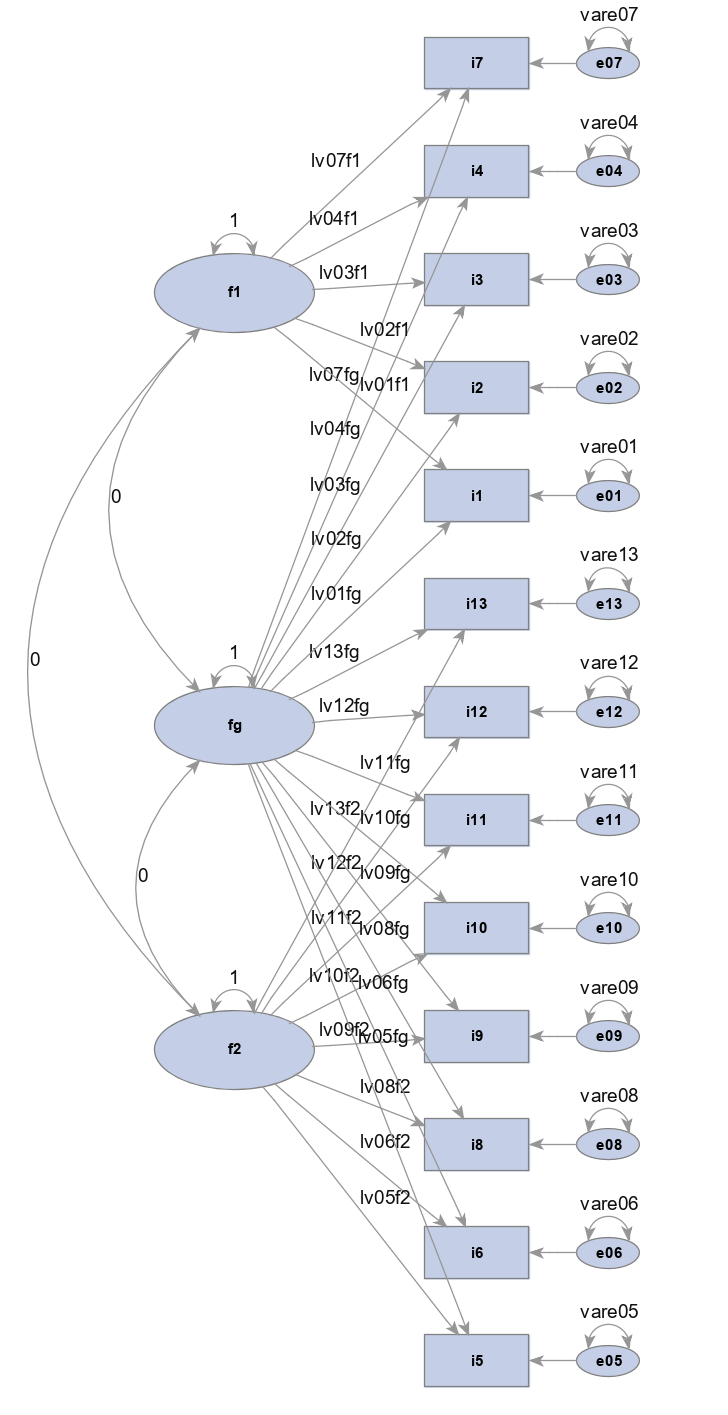


As with the second-order confirmatory factor analysis model, this bifactor model studied PD1 and PD2

The latent factor fg represents global factor for FACIT-F scale and f1 and f2 represent group/nuisance factors (which should be interpreted as residuals relative to the general factor fg)

FACIT-Fatigue: Functional Assessment of Chronic Illness Therapy-Fatigue; PD1/2: Pooled Data 1/2

APPENDIX 2D

## Figure S5 FACIT-Fatigue conceptual framework


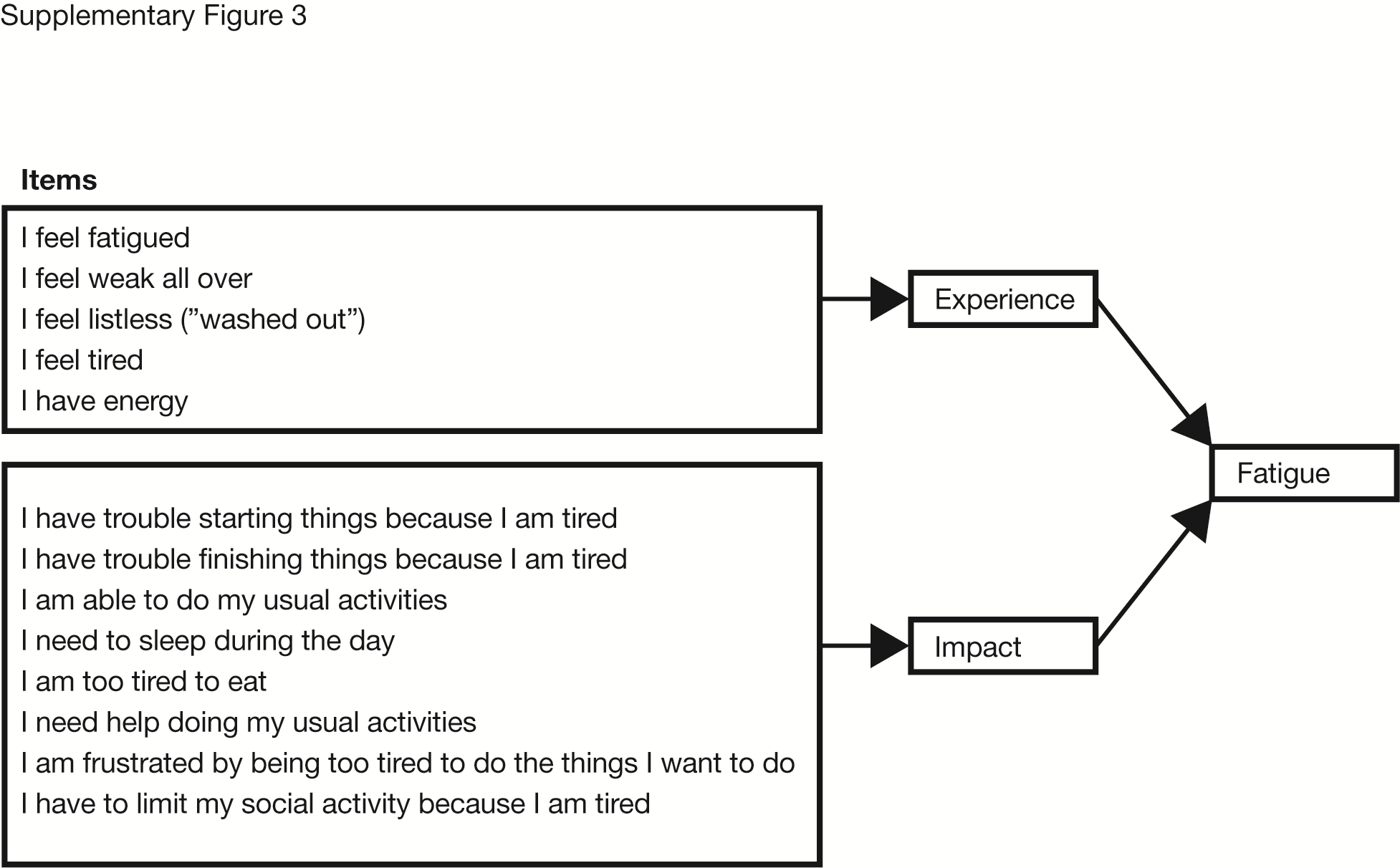


FACIT-Fatigue: Functional Assessment of Chronic Illness Therapy-Fatigue
