# Supplementary material for: Content validity and psychometric evaluation of Functional Assessment of Chronic Illness Therapy-Fatigue in patients with psoriatic arthritis
Source: J Patient Rep Outcomes. 2019 May 20;3:30. doi: 10.1186/s41687-019-0115-4 (PMC6527714; doi:10.1186/s41687-019-0115-4)
Supplement: Supplementary file 3 — Appendix 3: Test/retest reliability of FACIT-Fatigue in patients with PsA. (DOCX 12.4 kb) [file 41687_2019_115_MOESM3_ESM.docx]

ADDITIONAL FILE 3

APPENDIX 3

## Table S1 Test/retest reliability of FACIT-Fatigue in patients with PsA

|  | **ICC estimation of FACIT-Fatigue** | | |
| --- | --- | --- | --- |
|  | **Experience domain** | **Impact domain** | **Total score** |
| Pooled baseline and Month 1 data  (OPAL Broaden and OPAL Beyond) | 0.80 | 0.83 | 0.83 |
| OPAL Broaden | 0.78 | 0.78 | 0.79 |
| OPAL Beyond | 0.81 | 0.87 | 0.87 |

An ICC of ≥ 0.7 was considered acceptable [27]

FACIT-Fatigue: Functional Assessment of Chronic Illness Therapy-Fatigue; ICC: Intraclass Correlation Coefficient; PsA: psoriatic arthritis
